# Robust seed germination prediction using deep learning and RGB image data

**DOI:** 10.1038/s41598-021-01712-6

**Published:** 2021-11-11

**Authors:** Yuval Nehoshtan, Elad Carmon, Omer Yaniv, Sharon Ayal, Or Rotem

**Affiliations:** Seed-X LTD, 5691000 Magshimim, Israel

**Keywords:** Plant breeding, Plant development, High-throughput screening, Isolation, separation and purification

## Abstract

Achieving seed germination quality standards poses a real challenge to seed companies as they are compelled to abide by strict certification rules, while having only partial seed separation solutions at their disposal. This discrepancy results with wasteful disqualification of seed lots holding considerable amounts of good seeds and further translates to financial losses and supply chain insecurity. Here, we present the first-ever generic germination prediction technology that is based on deep learning and RGB image data and facilitates seed classification by seed germinability and usability, two facets of germination fate. We show technology competence to render dozens of disqualified seed lots of seven vegetable crops, representing different genetics and production pipelines, industrially appropriate, and to adequately classify lots by utilizing available crop-level image data, instead of lot-specific data. These achievements constitute a major milestone in the deployment of this technology for industrial seed sorting by germination fate for multiple crops.

## Introduction

Germination is a major seed quality attribute, as it directly impacts produce yield and quality. It is essentially a composite of two sub-attributes: seed germinability (seed ability to germinate) and seed usability (seed capacity to uphold a vigorous seedling emergence, interchangeably coined germination vigor)^1^. Seed lots designated for agricultural production are expected to meet certain germination standards, e.g., ≥ 90% usability for premium vegetable seed lots that mark an upper limit qualification standard (personal communication with vegetable seed companies). However, germination within an isogenic seed lot and even amongst sibling seeds is fairly heterogeneous, primarily due to plant innate seed development program that bet-hedges progeny succession by installing seeds with various maturity and dormancy levels^2^. Seed damaging during seed production, harvesting and post-harvest processing, and sub-optimal storage conditions lead to seed deterioration and dying and to emergence of abnormal seedlings, also contributing to reduction in usable seed portions^3^.

Seed lots are hence subjected to sampling-based germination quality control prior to selling or sowing^4^. Lots meeting germination standards are approved for sowing, but lots bearing lower ratios undergo separation processes aiming to raise seed germination to acceptable rates. These processes are based on limited correlations between physical parameters (seed size, weight or color) and seed germination fate, and are exerted by mechanical separators (*e.g.*, gravity tables, liquid density separation systems or sifter machines, segregating seeds by defined seed weight or size, respectively( or optical sorting machines (sorting by size and color criteria)^5–9^. These processes segregate the seeds by pre-defined parameters and fixed gates and very often they result with insufficient separation. Therefore, in practice, a separation effort is multi-step, comprising different, consecutive separation processes and accompanied by a considerable loss of usable seeds. Recalcitrant lots failing to meet germination rates by the end of the procedure are disqualified or priced low (Personal communication with seed producers). Collectively, these separation procedures are wasteful, time consuming and risky, as they may after all result with flawed seed delivery. On top of that, seed companies are often required to mitigate non-uniform seed usability by applying germination priming, a controlled imbibition process that invigorates aged and dormant seeds, such that primed seeds germinate vigorously and uniformly, at the expense of reduced shelf life (conservatively 6–12 months, as opposed to several years for untreated seeds at proper storage conditions)^10^. Moreover, the procedure does not expel dead or defective seeds.

Owing to the advancements made in computer vision, artificial intelligence (AI) and computation power, numerous attempts have been made in recent years to streamline seed (and grain) quality determination by developing automated, non-destructive, predictors operating per seed, rather than per statistical sample, for gauging seed quality attributes, such as seed health and safety, physical and genetic purity, chemical composition and germination^11–13^. These prediction tools are based on digital imaging and machine or deep learning, two AI disciplines to extrapolate probabilities based on a priori training of different classifying algorithms (*i.e.*, classifiers) using representation of the sampling space (known as the training set)^14^. The two learning approaches differ in the algorithms employed and the narrowing of machine learning to particular measurable features. Deep learning utilizes artificial neural network algorithms and does not delimit analysis to specific predefined parameters^14^. Color imaging (termed RGB for assigning Red, Green and Blue colors per pixel), suitable for collecting morphological data (*e.g*., color, size, shape and surface texture), was adopted for analyzing physical integrity^11–13^. Given the apparent deficiency of RGB imaging in tracking latent attributes as germination or chemical composition, spectroscopies of invisible wavelengths were employed to visualize seed anatomy (X-ray or magnetic resonance) or to monitor specific seed components (infrared or fluorescence imaging)^11–13^.

Mounted on low-throughput quality control instruments or on high-throughput sorting machines, such optical analytic tools are already applied industrially, making use of RGB-only or multispectral imaging and machine learning analysis to monitor mostly physical uniformity and purity (detection of seed dockage and damaged or irregular seeds), but also fungal contamination and amount of certain seed components (*e.g.*, protein, oil and starch)^15–18^. Still, many quality attributes of industrial value, such as genetic makeup, residual toxicity and organoleptic properties (in grains), are not supported by those commercial products. Determination of these attributes depends on experimental, destructive assays run on statistical samples that cannot be leveraged to seed separation in real time. Likewise, there is no commercial optical solution for germination prediction. To qualify seed lots, the seed industry is still entirely dependent on the conventional separation procedures and on seed priming with their acknowledged shortcomings^5–9^. X-ray radiography coupled with deep learning classification has been industrially adopted in recent years for sorting out incompletely developed or damaged seeds within tomato and pepper seed lots, already undergoing separation and priming procedures, whereas non-primed seed lots do not present the needed discriminative anatomy^8^. Still, utilization of this sorting process for additional crops is being investigated^19–21^. Infrared or fluorescence imaging paired with machine learning have been sporadically examined across the last two decades for germination prediction of non-primed seeds^22–30^. These trials were designed in low complexity, with only a few seed lots included in each trial, serving merely as a proof of concept. They inclusively demonstrated varying competencies to distinguish between germinating and naturally non-germinating or heat-killed seeds and between vigorous and artificially aged-by-heat non-vigorous germinating seeds in several crops, including carrot, cabbage, castor, corn, Jatropha, melon, radish, soybean, spinach and tomato^22–30^. None of these efforts, however, has ripened to an operational tool, probably due to insufficient efficacy, reproducibility or robustness needed for real application. The high costs of high-resolution spectral detectors may also pose a market entry barrier for such products and inhibit their development^31^.

Here, we report the development of a viable, universal seed germination prediction tool that is based on deep learning and RGB imaging, contrary to general perception. Compared to the germination enrichment solutions available to date, the tool brings with it a paradigm shift: (i) Instead of following individual separation criteria and rigid gates, it weighs multiple image data parameters at once to produce a bespoke classifier that is based on and optimized for training set particularities, to categorize every seed by flexible, multivariate statistics. (ii) The tool predicts both germinability and usability of every seed inspected, revoking seed priming prerequisite. (iii) The tool is adaptive, with training data refining enabling to improve classifier performance. (iv) The classification model enables to tune output germinability, usability and seed loss rates to assure sorting output meets quality and quantity demands. Respectively, we show that the tool is highly robust, capable of improving germinability and usability rates of multiple, disqualified seed lots to ≥ 90% across a broad crop range and with reasonable seed losses. When adequately trained with a representative assortment of crop-level seeds, it is further able to properly classify new, untrained lots and varieties, differing from the seeds composing the training set in cultivation time and place, and genetics, potentializing seamless application, without recurring training phases. This tool constitutes a solid milestone in the process of developing the very first sorting machine that addresses germination fate per se, with an aim to provide the seed industry with a reliable and consistent solution that outperforms the preparatory procedures applied today with a single sorting event, based on the germinability and usability probabilities of every seed evaluated.

## Results

### Prediction of seed germination by RGB image analysis is applicable across a broad phylogeny

**Figure 1:**
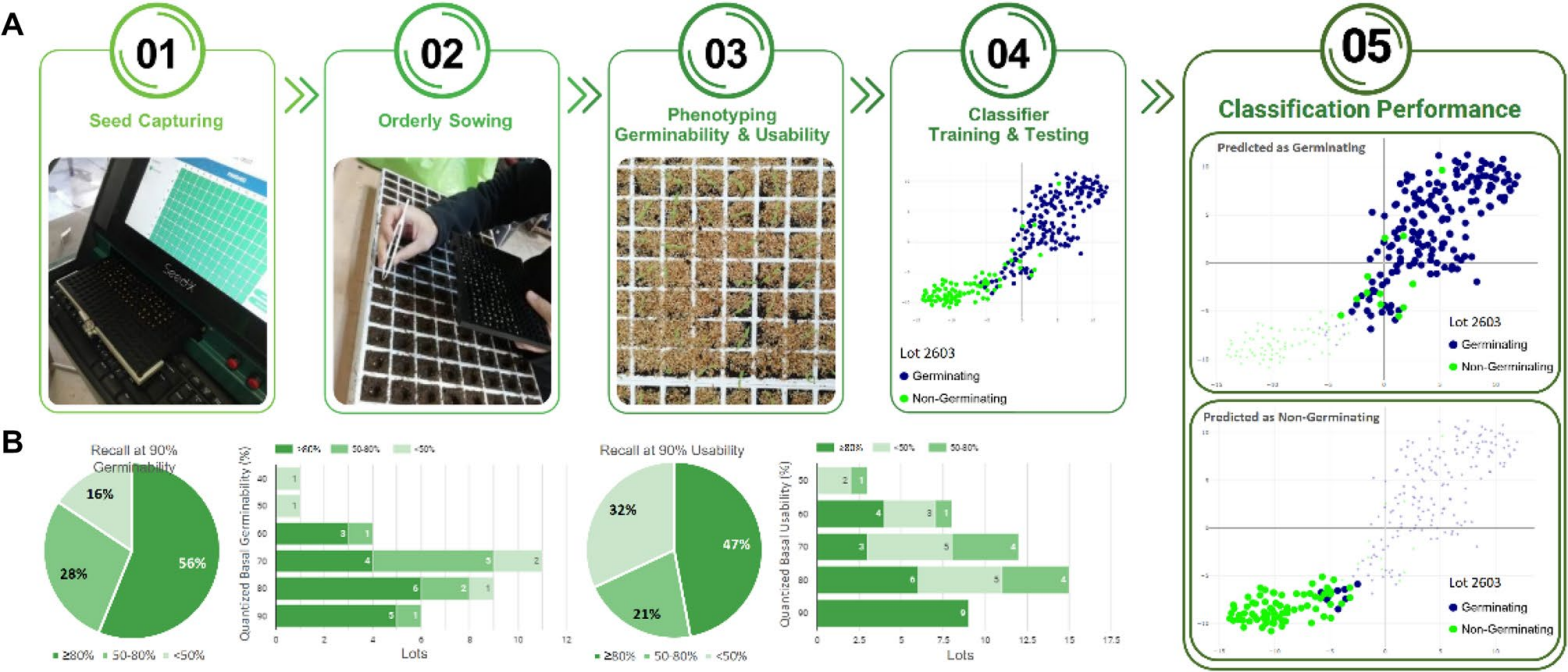
Germinability and usability prediction scheme and results. (**A**) The experimental process consisted of five steps: (1.) Seed-by-seed capturing of lot samples by GeNee Detect, (2.) orderly sowing, (3.) germinability and usability phenotyping, (4.) classifier training with 90% of the image and phenotypic data and classifier testing with the 10% left-out data, and (5) determining classification performance via the percentile bootstrap method and extraction of the average precision, recall at 90% precision and precision at 80% recall measures. The scatter plots at 4. and 5. visualize lot 2603 germinability prediction outputs. A scatter plot is a 2D projection of the multi-dimensional seed variation assigned by the classifier. Each dot represents a single seed and the closer the dots are, the more similar the seeds are. Here, dot colors represent actual phenotypes. At 4., seed dispersal resulted in an apparent, although incomplete, spatial separation between the blue germinating and green non-germinating test set seeds. At 5., setting a 90% precision ratio (upper plot, bold seeds predicted as germinating) resulted in a corresponding 98% recall, with 2% of the germinating seeds mistakenly associated with the seeds predicted as non-germinating (lower plot, bold seeds). (**B**) Pie charts: all 32 germinability and 47 usability predictions achieved 90% precision, but with various recalls. 56% and 47% of the predictions, respectively (51% in total), were ascribed with high (≥ 80%) recall. Bars: high recall was achieved across a broad basal germinability or usability, as low as 59% and 57%, respectively (here both quantized to 60%). Quantized rates (%) encompass ± 5% values. Scatter plots were generated by Plotly.js version 2.5.1: https://github.com/plotly/plotly.js.

A straight-forward approach would be prediction of seed germinability and usability by classifiers trained with samples of a composite of lots, as it would both expand the training set with morphological variation that can be beneficial for the training process and circumvent the need for classifier training per lot. Accordingly, forty-seven disqualified seed lots of 12 seed companies, clustered to 29 varieties, pertaining to 7 vegetable crops and ascribed with various, below 90% germinability or usability rates were sampled by hundreds to a few thousands of seeds each (Table S1). Every seed was color imaged and then assayed for germinability (germination onset at an early time point) and usability (seedling vigor at a later time point). Following a stratified train-test split, 90% of the images were clustered based on their respective germination performance to “germinating” or “non-germinating” clusters for germinability and “usable”, “abnormal”, “delayed” or “null germination” clusters for seed usability. These clusters were used to train two classifiers to distinguish between the different germinability or usability performances of all lots. The 10% left out data were used to test the performance of the trained classifiers per lot. Test seeds of each lot were scored with different probabilities to bring about each of the germinability and usability phenotypes and assigned with the most probable one per criterion. Images were then labeled with the actual performances and following the percentile bootstrap method, 10,000 independent output samples were generated. Two measures were extracted per given score threshold for each lot: the recall and the precision. The former indicates “germinating” or “usable” seed recovery rate [True Positives/(True Positives + False Negatives)] and the latter–“germinating” or “usable” seed purity rate [True Positives/(True Positives + False Positives)] amongst the seeds predicted as such. Average Precision (AP), the area under the precision *vs.* recall curve (Fig. S1A), was extracted per lot, being a general measure of the improvement granted by the prediction model over the baseline rate, that can also be considered as the AP of a null model that does not discriminate between the seeds. Two complementing score thresholds were further selected for each lot: one resulting with 90% precision (*i.e.*, 90% germinability or usability) and another leading to 80% recall (up to 20% loss of germinating or usable seeds). 90% precision was selected as it matches the highest germination standards applied in the seed industry today, for premium vegetable seeds. 80% recall was set based on our appreciation that 20% seed loss is the upper acceptance limit for premium vegetable seed lots during seed separation (personal communication with vegetable seed companies). Classification merit to reach or approximate 90% precision while retaining at least 80% recall was assessed by the reciprocal recall and precision values. Mean values, based on the 10,000 bootstrap sampling iterations, are reported with their standard deviation and 95% CI at Table S1. The entire process is illustrated in Fig. 1.

**Figure 2:**
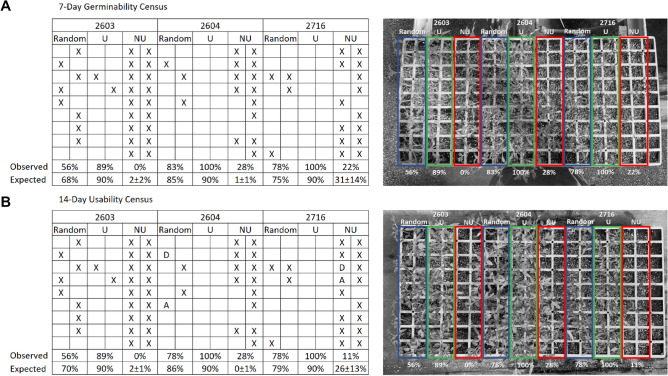
Sorting of three tomato seed lots by usability. Lots 2603, 2604 and 2716, pertaining to three distinct varieties, were sorted by their usability predictions and phenotyped for germinability and usability 7- and 14-days post sowing, respectively. Phenotyping data on the left correspond with the germination status captured on the right. A random selection, reflecting basal germinability or usability rates was compared to putative usable and non-usable portions, each represented by 18 seeds. Observed germinability and usability rates were compared to the expected ones (data taken form Table S1). Differences between the two are likely to result from the low sampling size. Still, germinability and usability were markedly enhanced to the expected rates. Random–random selection (framed in blue rectangle on the right); U-putative usable (framed in green rectangle on the right); NU-putative non-usable (framed in red rectangle on the right); X-non-germinating seed; A-abnormal seedling; D-delayed seedling.

**Figure 3:**
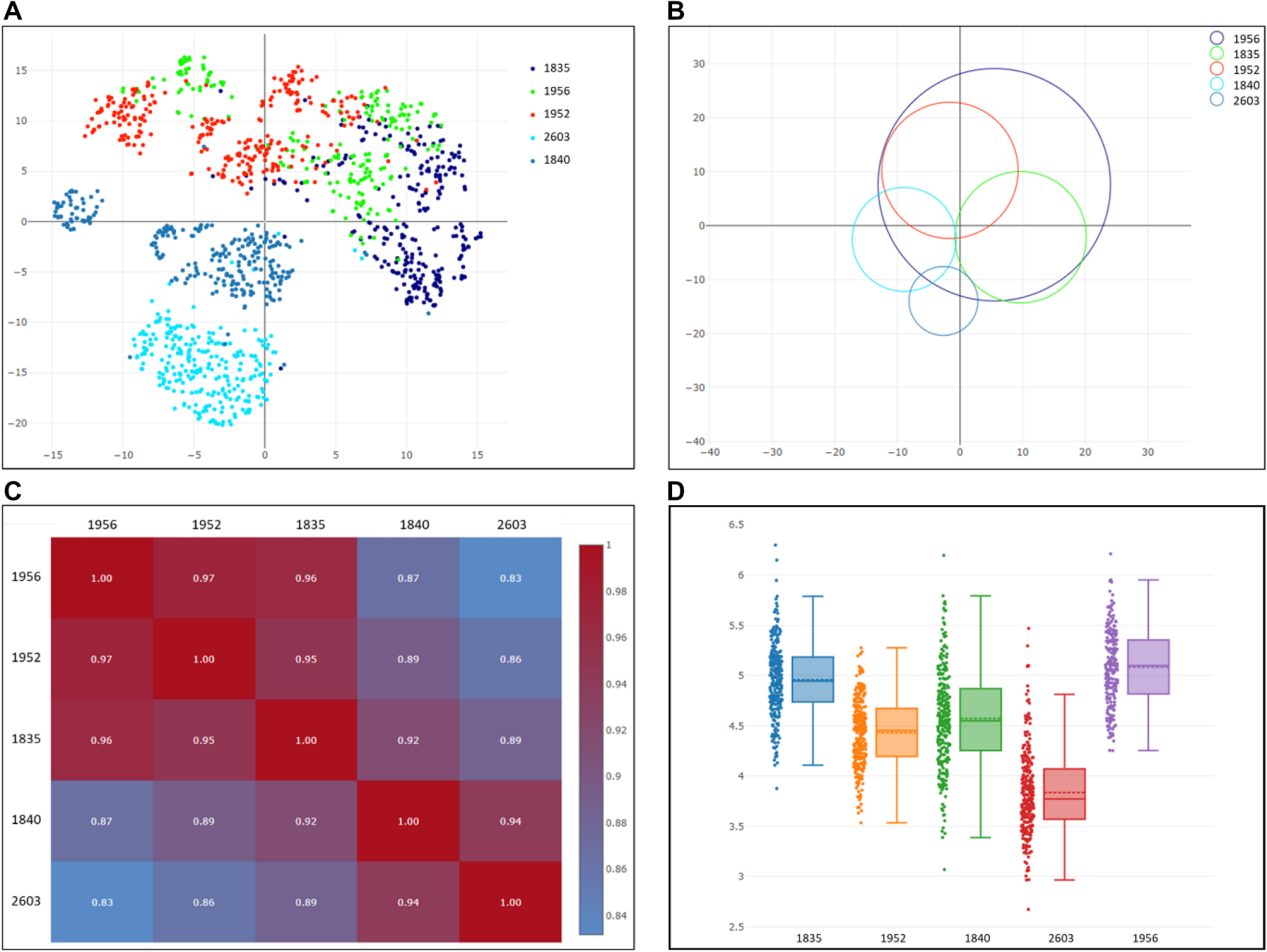
Morphological heterogeneity within and between five isogenic seed lots. Seed images of lots 1835, 1840, 1952, 1956 and 2603, all of variety 21, were assigned to train (90% of the data) and test (10% left-out data) a classifier to distinguish between them. Intra-lot uniformity and inter-lot similarity assigned by the classifier to the test set are conveyed by (**A**) a scatter plot, where each dot represents a given seed and lot positioning and dispersal reflects its level of uniformity and relatedness to other lots; (**B**) a bubble chart, a lot-inclusive presentation, where bubble diameter is proportional to intra-lot variance and position correlates with inter-lot similarity; (**C**) a similarity matrix, where overall similarity between the lots is denoted numerically in 0–1 scale (0 indicates no relatedness between the lots and 1—an exact match). Cell colors correlate with the numbers, according to the color bar on the right; (**D**) seed area box plot, one of numerous morphological characteristics reckoned by the classifier. (**A**–**C**) portray the partition of the isogenic lots into two similarity groups, one of 1840 and 2603 and another of 1835, 1952 and 1956, and the uneven lot uniformity that ranges between 2603, the most uniform lot to 1956, the least uniform one. Seed area distribution does not correlate with classifier findings that are based on multiple morphological measures. Plots were generated by Plotly.js version 2.5.1: https://github.com/plotly/plotly.js.

The APs across 79 (32 germinability and 47 usability) classifications were increased compared to the baseline rate by 1.27 ± 0.20 fold, with a mean value of 0.91 ± 0.06. Per lot, germinability and usability predictions yielded similar APs (1.00–1.09 fold-change), except for lots 2760, 2793 and 2799 that produced 1.15, 1.22 and 1.23 germinability over usability fold-change, respectively. Lot 2799, in particular, had a satisfactory basal germinability rate of 94% but a basal usability rate of 79%, reflecting the difference between these two facets of germination. All classifications achieved 90% precision. Using the usability prediction model, three tomato seed lots of distinct varieties—2603, 2604 and 2716—were sorted and then sown and phenotyped after 7 and 14 days, demonstrating 33%, 17% and 22% germinability and 33%, 22% and 22% usability rate improvements, respectively (Fig. 2). Recall at 90% precision was found less congruent per lot than AP, with 22% (7 of 32) lots showing superior germinability prediction (1.24–24.43 fold-change), 56% (18 of 32) lots presenting similar values for germinability and usability (1.00–1.09 fold-change) and 22% (7 of 32) having greater usability recall (1.12–7.55 fold-change). The remaining 15 lots were only phenotyped for seed usability. Inferior usability prediction probably resulted from insufficient representation of non-usable phenotypes in the training set. Inferior germinability prediction was brought about by premature germination census that missed germinating seeds, as all these varieties had basal usability rate higher than basal germination rate. Germinability and usability models generated 74% ± 28% and 67% ± 31% recalls at 90% precision, respectively. 56% (18 of 32) of germinability predictions and 47% (22 of 47) of usability predictions (51% in total) yielded ≥ 80% recall at 90% precision and all but one having precisely 80% recall were further amenable for precision improvement beyond 90% by reducing the recall to 80%. These predictions account for different lots of the *Solanaceae* tomato (16 lots, 10 varieties) and pepper (7 lots, 5 varieties), the cucurbit watermelon (1 lot) and the cruciferous cabbage (1 lot) and cauliflower (1 lot). Tomato and pepper are the only crops represented by multiple seed companies. 5 of 6 companies representing tomato and 4 of 6 companies representing pepper, and a total of 9 of the 12 companies involved in this study, produced lots that met the ≥ 80% recall criterion, indicating the ability of the methodology to cope with different breeding programs and different production pipelines. 24% (19 of 79) and 25% (20 of 79) of the classifications provided moderate (50%-80%) to low (< 50%) recalls at 90% precision, respectively. These recalls are associated with lots of varieties already reaching ≥ 80% recall, other varieties of the same crops and two more crops: the herb celery and the monocot geophyte onion. The fact that different varieties of the same crop and especially different lots of the same variety (varieties 4, 5, 15, 16, 24, 25 and 26) presented different classification performances illustrates the high level of heterogeneity embedded in seed morphology between and within varieties (Fig. 3), affected not only by genetics, but also by external factors across the production pipeline (plant growth, seed harvesting, processing and storage). Although deemed unsatisfactory due to insufficient recall, prediction performance for these lots may be improved by enriching the training sets with higher representation of seeds from these lots that will afford better covering of their seed variance and underpinning of the differences between their phenotypic groups.

Altogether, the results demonstrate the applicability of this inclusive approach to sift seed lots and set ≥ 90% seed germinability or usability across a broad vegetable range, encompassing monocots and various dicots. With more than half of the experimented predictions meeting the ≥ 80% recall criterion, classifier training with seeds sampled from a composite of crops, varieties and lots was a viable approach, but evidently, training set composition was inadequate for all use-cases. Plausibly, better representation of some seed morphologies can be achieved not only by collecting more data directly from the challenging lots but also by incorporating data from other, analogous lots. The possibility of replacing data production with already available, compensatory data is especially intriguing as ad hoc data production may be demanding and throughput-limiting in some industrial settings.

### Training set expansion with crop-level data counteracts reduction in ad hoc data production

We premised data production per unmet lot might be reduced or even replaced by a training set comprising data from analogous lots that would represent satisfactory seed level diversity. Accordingly, the more relevant data incorporated the more precise seed representation becomes up to a point where standardized and streamlined classification is achieved per variety, per crop and, possibly, beyond those, without ad hoc data production. To test this, we focused on tomato, the most diverse crop in our seed collection, represented by 26 lots and 14 varieties from 6 different seed companies (Table S1). First, an all-*versus*-all image-based similarity matrix (Table S2) was generated and 6 lots across the matrix, representing different similarity groups, were assigned as test lots. They were composed of recurrent (1837, 2509, 2603, 2717, 2815) or unique (3012) varieties, compared to the remaining 20 lots, and similarly divided between two seed companies. The remaining 20 lots constructed four Training Sets (TS1-4), representing gradual data diversification, generated using image and phenotypic data of 6, 10, 16 and 20 tomato seed lots of 6, 9, 11 and 13 distinct tomato varieties, respectively, in an additive manner (Table S3). TS1-4 were assembled based on similarity to the test lots, starting with the most dissimilar lots at TS1 (similarity score cutoff: ≤ 0.86) and incorporating increasingly similar lots along TS2-4 (TS2 ≤ 0.9; TS3 ≤ 0.96; TS4 ≤ 1.0), imitating data accumulation scenario up to redundancy (Table S2). Noteworthy, lots 2509, 2603 and 2717 were found equally or more similar to genetically disparate lots than to genetically identical lots, demonstrating, again, the gravity of environmental influences on seed shape. The six test lots were subjected to 19 independent usability predictions, based on a training matrix composed of TS0-4 (“TS0”—where no auxiliary usability data were used) and 0, 600, 1000 or 1400 images of the given test lot that were split in a stratified fashion to properly represent the different usability phenotypes and not included in the test sets. Of 20 possible combinations, all but ‘TS0 and 0 lot-specific images’ (*i.e.*, no usability training data) were examined. AP, ‘recall at 90% precision’ and ‘precision at 80% recall’ measures were extracted per prediction model and all measures collectively reflected the changes, brought about by expanding auxiliary (TSs) and/or lot-specific data (Table S4).

**Figure 4:**
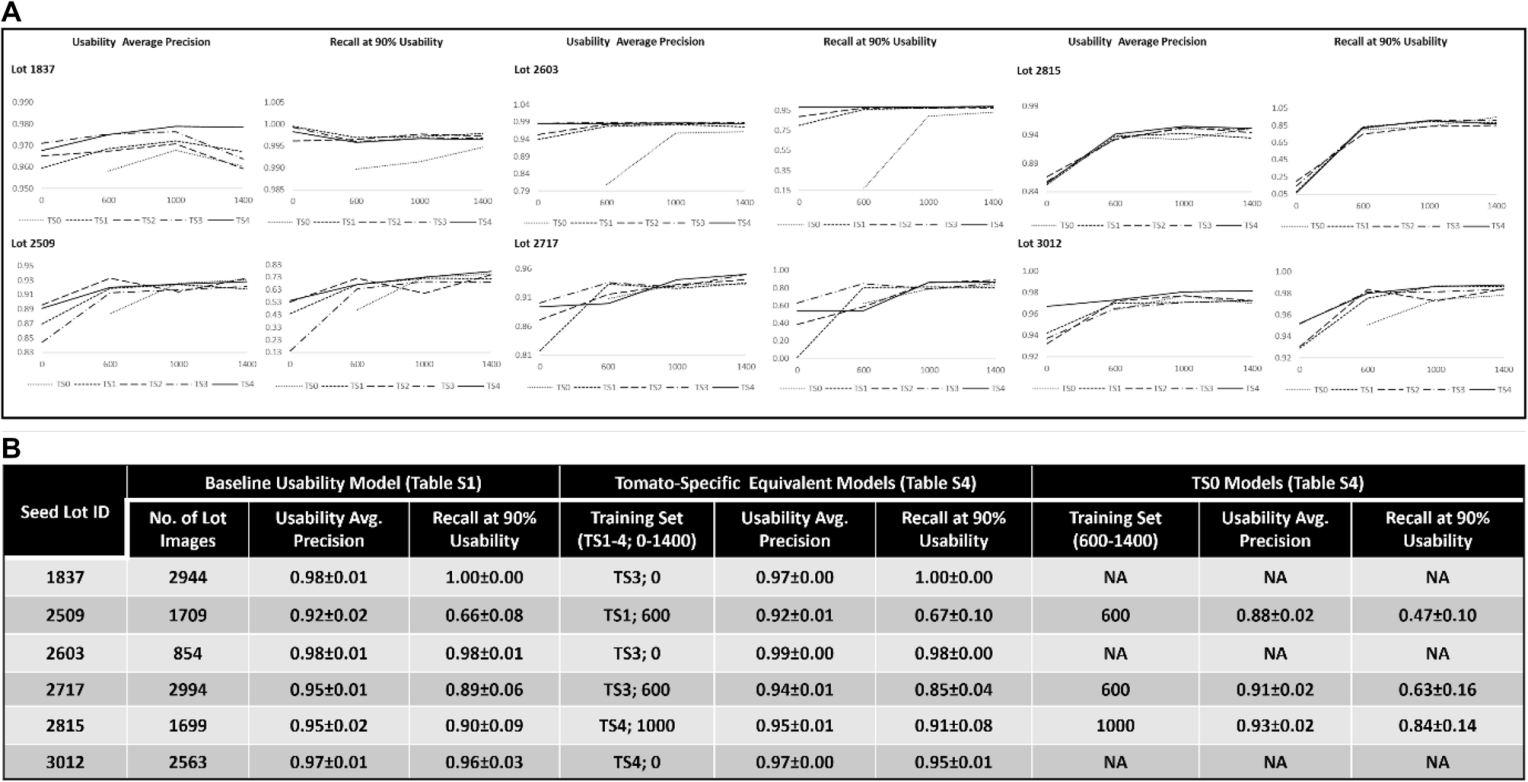
Counterbalancing lot-specific data reduction with crop-level auxiliary data. Usability of six tomato seed lots was predicted by 19 separate classifiers that had been trained with a matrix of accumulative auxiliary training sets of 0, 6, 10, 16 or 20 different tomato seed lots (respectively, TS0-4) combined with lot-specific data (0, 600, 1000 or 1400 images, weighted by phenotypes). All combinations but ‘TS0 and 0’ (no training data) were trained with. (**A**) ‘Usability average precision’ and ‘recall at 90% usability' were generally improved by training set diversification, but in some cases, this led to a decrease in classification performance, probably due to imbalanced training set expansion and the stochasticity of the training process (see text for more details). (**B**) A comparison between the baseline usability model and the Tomato-specific equivalent models, trained with crop-level auxiliary data, demonstrated the competence of the latter to generate similar performances with significantly less-up to exclusion—of specific data. TS0 models, trained by the lot-specific component of the ‘tomato-specific equivalent model’ training sets generated inferior performances, reflecting the added-value of the auxiliary data. NA-non-applicable, where TS1-4 equivalent models used no specific data.

Ideally, changes were expected to follow a saturation model or parts of it, under the premise that the more data the better until saturation is attained. In practice, additional incoherent patterns were obtained, involving occasional declines in prediction performance (Fig. 4A). Those may be attributed to patchy representation of the actual test set diversity in the training sets, across both TS1-4 and the 6 lot-specific image data, that led to imbalanced training set expansion, model overfitting to nuances and to suboptimal model performance. The stochastic progress of the training process may have also contributed to mild declines between adjacent training sets and explain, for instance, lot 1837 classification dynamics. Still, the training matrix enabled us to evaluate auxiliary data impact on prediction performance. Training by extensive lot-specific data (1000 and 1400 images) alone outperformed training by auxiliary data alone in four lots (2509, 2717, 2815, 3012). In lot 2603 the auxiliary data outcompeted the specific data and in lot 1837—performances were equivalent. Composites of both auxiliary and lot-specific data generated the best performances in all lots but 2815, where best performance was obtained with 1400 lot-specific images and incorporation of auxiliary data had a detrimental effect. Noteworthy, composites of TS3 or TS4 with 600 or 1000 images of lot 2815 were superior to the image data alone. Comparing performances attained with the tomato-specific training set (Table S4) and the ones produced with the global training set (Table S1), the former yielded for all six test lots predictions similar to those attained with the baseline usability models, but with far less (2509, 2717, 2815) to none (1837, 2603, 3012) lot-specific data, which were unable by themselves to reproduce those performances (Fig. 4B). Specific data of 2509 and 3012 (600 images, supporting TS2 and TS1, respectively) led to superior predictions by the tomato-specific model, notably 0.66 ± 0.07 to 0.72 ± 0.07 and 0.96 ± 0.03 to 0.98 ± 0.01 ‘recall at 90% usability’ improvements, in spite of 2.8- and 4.3-fold more data utilized by the baseline model, respectively. Taken together, these observations illustrate the advantage of the tomato-specific auxiliary data over the global training set not only in replacing specific data but also in improving classification.

A closer look into the predictions generated by the auxiliary TSs show that lots 1837, 2603 and 3012 were assigned with ≥ 80% recall at 90% usability already with the rudimentary auxiliary training set (TS1 with 0 images of those lots), composed of the most dissimilar seed lots (Table S2). More expanded TSs improved prediction performance (TS4 for 3012) or even exceeded the prediction performance of the model relying on training by extensive lot-specific data (TS3 for 2603). As already noted, the variety of lot 3012 was not represented in TS1-4, hence its usability prediction was entirely dependent on other varieties. When 600 images of 3012 were added to TS4, a 0.95 ± 0.03 to 0.98 ± 0.01 ‘recall at 90% precision’ improvement was achieved and was not met by the 600 images alone. Lots 1837 and 2603 already presented superb classification performance without specific data (1837—0.97, 1.00 and 2603—0.99, 0.98 AP and recall, respectively) and addition of which or data of more similar seeds in expanded TSs did not improve classification. With the auxiliary data alone, the remaining lots 2509, 2717 and 2815 did not reach 80% recall at 90% usability and their classification performances were inferior to the ones generated with 1000 and 1400 lot images. Evidently, the high similarity between lot 2509 and TS3-4 lots 2505, 2519, 2605 and the isogenic 2914 (Table S2) did not counterbalance 2509 data dismissal. Classification of lot 2815 was practically indifferent to the auxiliary data alone, producing only marginal recalls through TS1-4, although the genetically identical—but visually dissimilar (Table S2)—lot 2605 had been included in TS4 and 11 more lots (5 varieties) coming from the same seed company had been embedded in TS2-3. Introducing specific data drove performance improvement: TS4 and 1400 images of lot 2509 and TS2 and 1400 images of lot 2717 supported the best outputs for these lots. Auxiliary training set failed to meet the performance produced by 1400 images of lot 2815 alone, but combined with 600 and 1000 images, TS1, TS3 and TS4 led to improved classification.

Overall, these results support our premise that training set expansion with data coming from other lots and varieties of different companies can be used to reduce and, in some cases, replace specific data production for new lots and even for unmet varieties (*i.e.*, lot 2603), while retaining or even improving classification performance. This exercise further pinpoints that training set expansion should be fine-tuned cautiously rather than inflated in an unrestrained manner, as unbalanced representation of the diversity is inevitable, and, as shown, may lead to a decrease in classification performance. Further, lot 2815 demonstrates that some lots may be more challenging than others to be represented by other lots, due to external impacts.

## Discussion

The industrialized agriculture of the 21^st^-century is still being challenged by seed germination^1,3^. With only partial solutions available to date to determine seed germination fate^5–9^, the seed industry has been settling on sub-optimal germination standards, culminating to ≥ 90% in the case of premium vegetable seeds that are expected to attain superior performances. This translates to severe resource waste and yield loss, in an era where beyond financial considerations, the cruciality of sustainability and food security are well-recognized^32^. Our proposed methodology to employ optics for efficient and non-detrimental seed germination prediction proceeds earlier attempts, but contrary to those, ours utilizes color imaging and neural network classification. We employed it to robustly enrich seed germinability and usability, two different aspects of germination, to ≥ 90% precision, in a cohort of 7 different crops, spread across a broad phylogeny and represented by at least two lots (Table S1), bred and produced by 12 different companies. This new capability is expected, once installed on a seed sorter, to enable seed companies for the first time to segregate seeds of multiple crops based on their germination potential in high-fidelity, in a single separation process and without compulsory seed priming, and that way both streamline seed germination enrichment process and render lots that with contemporary separation techniques would have failed enrichment attempts, such as the lots used in this study, industrially appropriate. Unfortunately, we know only for a minority of the lots the basis for mal-germination (data courteously provided by the seed producing companies), which was either genetics (lots 3012 and 3013), production (lots 2982 and 2983) or extended storage (2509). Those lots cover the possible circumstances leading to mal-germination and classification of which met the ≥ 90% precision and ≥ 80% recall criteria, demonstrating method competence to cope with different grounds for mal-germination.

Precision and recall are in tradeoff and therefore, retaining satisfactory recall and precision requires very accurate classification (Fig. S1). In real practice, precision and recall can be easily modulated, based on the prediction model, to provide a sorted fraction that meets seed company quantity and quality demands (Fig. S1B). In our case-study, ≥ 90% precision and ≥ 80% recall were given specific attention, being stringent qualification criteria, applied on premium vegetable seed lots. 51% (40 of 79) of the classifications relying on the global training set (Table S1) and 71% (68 of 96) of the classifications of tomato lots utilizing tomato-specific training sets (Table S4) met the ≥ 90% precision and ≥ 80% recall criteria, demonstrating that those standards are achievable with this methodology at least for 5 (tomato, pepper, watermelon, cabbage and cauliflower) of the 7 experimented crops. Classifications not reaching those standards presumably derived from intra-lot heterogeneity that was not covered well-enough by the training sets. Conspicuously, lots of all 7 crops (with the addition of celery and onion), part of which of varieties that already met the precision and recall criteria, failed to meet them and exhibited the magnitude of external influences on seed variance, in a manner that was not limited to a given crop or variety. This motivated us to experiment in tomato the impact of training set enrichment with relevant data, from the same lot (with increasing numbers of images) and from related ones, of the same variety or of other varieties of the same crop (TS1-4) (Fig. 4). The results demonstrated the usefulness of auxiliary data not only to salvage lots, but also to reduce data production.

Classification of lot 3012 that experienced poor basal usability due to variety genetics relied on image data of genetically disparate seeds. This implies that different pathways leading to mal-germination converge into a consistent morphological footprint, making them comparable and eligible for cross-training. We postulate that such crop-specific training set diversification would similarly enable us to mitigate classification in celery and onion, two crops that were represented by only 2 lots of a single variety each and were not meeting the ≥ 80% criterion, and, in a broader sense, facilitate satisfactory germination prediction in crops not included in this study. Congruence between seed data of taxonomic hierarchies higher than crop species may also be considered, but this is beyond the scope of this work.

In this study we challenged our classification method to meet the highest industry germination standards. It is only to be expected that this methodology would ultimately enable us to set new, improved standards, by reaching higher precision and recall across multiple lots. This work further shows that beyond conventional physical uniformity analysis, RGB data can be exploited to elucidate seed germination fate by adopting high-end artificial neural network analytics. Contrary to spectroscopy methods that are based on optical properties of particular chemical entities, *e.g.*, germination prediction by measuring seed chlorophyll levels^22,28^, whose levels and association to the attribute in question may vary between crops, or to X-ray radiography that cannot be applied on any structure and therefore in the context of germination is applicable on primed seeds^8^, this method utilizes morphological data not limited by any defined physicochemical characteristics, making it a priori eligible for any seed of any crop. It remains to be determined which attributes additional to germination may be credibly classified with this data type, supported by the proper analytics.

## Methods

### Seed collection

Forty-seven samples of uncoated, processed seed lots of Cabbage, Cauliflower, Celery, Onion, Pepper, Tomato and Watermelon were received from 12 different seed companies (Table S1). They were kept until further use under cool and dry conditions and used in compliance with relevant national or international guidelines.

### Seed imaging, sowing and phenotyping of seed germinability and usability

Seeds were captured in high color resolution by Seed-X “GeNee Detect” platform (https://www.seed-x.com/product/detect/) as follows: First, seeds were positioned in an orderly fashion on GeNee Detect trays. Due to different seed size and color per crop, seeds of different crops were placed on different trays, as illustrated in Table 1.

**Table 1:** GeNee Detect trays used for seed capturing per crop in this study.

Crop	GeNee Detect tray
Cabbage	247-well silver tray
Cauliflower	247-well silver tray
Celery	247-well black tray
Onion	247-well silver tray
Pepper	247-well black tray
Tomato	247-well black tray
Watermelon	90-well black tray

Loaded trays were fed into GeNee Detect imaging chamber and captured by an IDS UI-3884LE-C-HQ-AF 6.41 Mpix RGB camera. Imaged seeds were sown on standard garden soil trays, while keeping seed order and covered with Vermiculite 2–3 mm. Trays were incubated in the dark for 2–3 days and then transferred to the greenhouse. Two germination censuses were conducted (Table 2). The first time point monitored timely germinability and the phenotypes collected were germinating or non-germinating seed. The second time point monitored seed usability and the phenotypes collected were of usable, abnormal, delayed, or null germination.

**Table 2:** Census timepoints for seed germinability (time point 1) and usability (time point 2) per crop.

Crop	Time point 1 (days)	Time point 2 (days)
Cabbage	7	14
Cauliflower	7	14
Celery	14	21
Onion	10	17
Pepper	10	17
Tomato	7	14
Watermelon	7	14

### Image processing

Imaged seeds were detected using global thresholding and connected component algorithm. The detected area of interest was cropped into a fixed size of 700X700 pixels.

### Germination classification by DCNN

Seed-X GeNee technology uses DCNNs^14^. The backbone architecture “SeedNet” is based on Inception^33^, trained on Seed-X proprietary database of millions of seed images, reflecting multiple crops, genetic backgrounds and seed production pipelines. A cross entropy loss^34^ is used to classify the different lines. The training process uses industry standard method of stochastic gradient descent (SGD)^35^ with learning rate of 0.01 and momentum of 0.9 to create pretrained weights. Other configurations and hyperparameters were tested but, ultimately, these hyperparameters were selected, as they provided the best performing weights across several different classification tasks based on accuracy. “SeedNet” contains a good seed representation thanks to the seed database size and diversity, and constitutes an optimized transfer learning^25^ starting point for general seed classification tasks.

### Training

For each specific germination dataset the basic architecture backbone was fine-tuned using transfer learning^36^ from the pretrained “SeedNet”. The training dataset was randomly split into training (90%) and test (10%). The training was performed using TensorFlow^37^. The optimization process used SGD, with an initial learning rate of 0.01 and a momentum of 0.9, using a cross-entropy loss function. Standard ImageNet augmentations^38^ were used during training.

### Evaluation

Model performance was evaluated using precision, recall and average precision measures. All values were reported with their 95% Confidence Intervals (CI), calculated by the percentile bootstrap method with 10,000 independent experiments^39^. For each seed image, a compressed representation was extracted from the trained neural network by seed embedding^40^. Using these analytical seed representations and averaged representations, distances between specific seeds and between lots were measured by the cosine similarity distance calculation method^41^. The distance matrix (Fig. 3C, Table S2) presents distance between lots. The representations were further reduced to lower dimensions using T-SNE^42^ for 2D scatter plotting (Figs. 1 and 3A). The bubble plot (Fig. 3B) was produced using seed representations, by creating for each lot a bubble positioned by its averaged representation with a radius relative to its variance.

## Supplementary Information

Supplementary Information 1.

Supplementary Information 2.
