# Supplementary material for: Robust seed germination prediction using deep learning and RGB image data
Source: Sci Rep. 2021 Nov 11;11:22030. doi: 10.1038/s41598-021-01712-6 (PMC8586350; doi:10.1038/s41598-021-01712-6)
Supplement: Supplementary file 2 — Supplementary Information 2. [file 41598_2021_1712_MOESM2_ESM.docx]

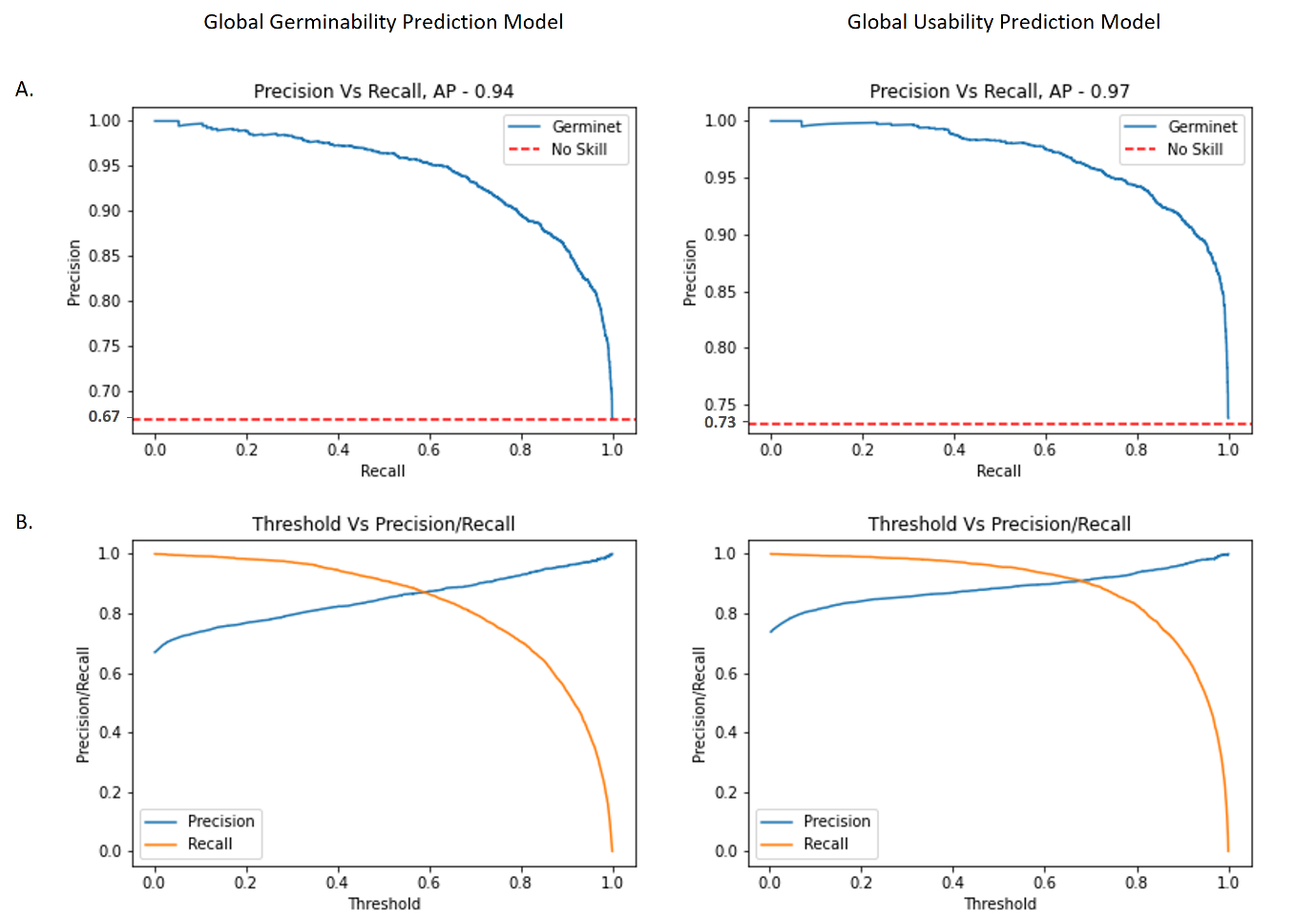


Figure S1. Precision and recall tradeoff. (A.) Precision *vs.* recall curves demonstrate the precision-recall tradeoffs obtained with the global germinability (left) and usability (right) prediction models. The higher precision is the lower recall becomes and *vice versa*. The area under the curve constitutes the average precision (AP). For simplicity, the Average Precision measures (APs) presented here (AP_Germinability_ - 0.94 and AP_Usability_ - 0.97) are the APs generated with the complete germinability and usability test sets. Table S1 provides specific APs for every lot, so prediction improvement over basal germinability and usability can be observed. The dashed line represents the baseline APs (AP_Basal germinability_ - 0.67 and AP_Basal usability_ - 0.73) accepted by null models (no-skill), classifiying germinating or usable seeds randomly. (B.) Threshold *vs.* precision/recall plots present the precision and recall tradeoffs obtained per score threshold (Threshold). Threshold selection enables to tune sorting performance and pre-determine the germination rate and the volume of the sorting output. Plots were generated by Matplotlib version 3.4.3: https://matplotlib.org/stable/citing.html.
